# Functional and aesthetic results of the Z-shaped and straight lower lip-splitting incision: a randomized clinical trial

**DOI:** 10.1038/s41598-024-63983-z

**Published:** 2024-08-12

**Authors:** Cheng Miao, Ying Zheng, Zhongkai Ma, Lu Bai, Guang Hong, Longjiang Li, Chunjie Li

**Affiliations:** 1 State Key Laboratory of Oral Diseases & National Center for Stomatology & National Clinical Research Center for Oral Diseases, West China Hospital of Stomatology, Sichuan University, Chengdu, China; 2 Department of Head and Neck Oncology, West China Hospital of Stomatology, Sichuan University, Chengdu, China; 3 Division for Globalization Initiative, Tohoku University Graduate School of Dentistry, Sendai, Japan; 4 Chengdu Sixth People’s Hospital, Chengdu, Sichuan China; 5 West China School of Nursing, Sichuan University, Chengdu, China

**Keywords:** Surgical wound, Lower lip-splitting incision, Reconstructive surgical procedures, Maxillofacial surgery, Cancer, Diseases, Oncology

## Abstract

The lip-splitting approach enables excellent access to all areas of the mouth and pharynx to remove tumors; however, traditional lower lip-splitting incisions produce an unsatisfactory scar. To achieve better functional and aesthetic results, we used a Z-shaped incision and compared the functional and aesthetic outcomes of the straight and Z-shaped incisions. Sixty patients who fulfilled the inclusion criteria were randomly divided into two groups and underwent lip-splitting between March 2021 and September 2023. Eventually, 77 patients were reviewed within 6 months and evaluated using the lip function assessment scale, patient and observer scar assessment scale, naïve observer scar assessment scale, and a clinical examination. The Z-shaped incision group performed better in terms of the lip pout movement at 3 months and in the subjective overall opinion, color, irregularity, and pigmentation at 6 months. The Z-shaped incision group had a lower incidence of notched vermilion. In conclusion, Z-shaped lower lip-splitting incisions have better functional and aesthetic outcomes than traditional straight incisions.

**Trial registration:** Public title: Difference between the effect of Z-shaped and vertical incisions of labiobuccal flap on the recovery of lower lip scars. Registration date: 09/03/2021. Registration number: ChiCTR2100044084. Registry URL: http://www.chictr.org.cn.

## Introduction

Three-dimensional extirpation of tumors with safe surgical margins while maintaining function and cosmesis after ablative surgical procedures is important in oral and maxillofacial surgery. However, nothing compares with the direct surgical field offered by a lower lip-splitting incision in terms of visual access, haptics, and vessel control^1^, especially when the lesion involves the maxilla, maxillary gingiva, and hard or soft palate^2^. However, this approach inevitably causes aesthetic and functional problems such as unsightly scars, vermilion notching, chin-pad contour loss, dwindled lip sensation, lip mobility, and oral commissure incontinence^3^.

The lower lip-splitting procedure has been widely used in oral and maxillofacial surgery since it was first described in mid-nineteenth century. It was later modified by Robson^4^, McGregor and McDonald^5^, Ramon^6^, Rassekh^7^, and Hayter^8^ in attempts to improve postoperative function and appearance. These modification strategies include using a relaxed skin tension line, hiding in the contour of the chin protuberance, reducing muscle fiber damage, and avoiding a straight-line incision across the lip. These are said to be effective, although there have been insufficient clinical trials. However, we believe that the best strategy among them is avoiding a straight-line incision across the lip because it limits wound contraction, which has been proven by fundamental research^9^. Therefore, we applied the Z-plasty technique to the lower lip-splitting incision and named it the Z-shaped incision. The biggest difference between the design of the Z-shaped incision and that of the straight-line incision is that the Z-shaped incision completely abandons the continuous straight line. This novel incision design includes the following: (1) the scar line is an elongated incision with several alterations in orientation that may reduce straight-line scar contracture; (2) it does not have a vertical incision at the vermilion that continues with the perioral incision, which may reduce the possibility of a notched vermilion and groove formation; (3) it has more landmarks for accurate wound closure, meaning that it does not require surgical marks on the skin, which may cause extra scarring; and (4) this design avoids the transection of the entire muscle bundle and accelerates wound healing. We believe that the novel Z-shaped lower lip-splitting incision may improve functional and aesthetic outcomes.

Therefore, we designed this randomized clinical trial (RCT) to compare the outcomes between the classical straight incision and our novel Z-shaped incision. This study is the first to show the postoperative recovery process of a lower lip-splitting incision with multiple time-point measurements, which may provide a potentially effective surgical approach for oral and maxillofacial oncology surgery.

## Methods

### Patients

**Figure 1:**
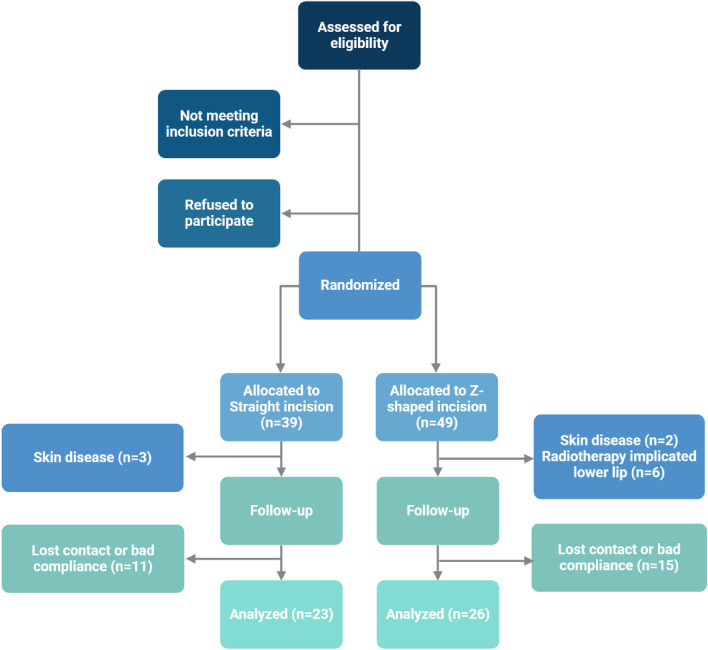
Work flow chart. Created with BioRender.com.

Between March 2021 and September 2023, patients were admitted to the Head and Neck Oncology Surgery Ward of the West China Hospital of Stomatology, Sichuan University, and 77 eligible patients were randomly enrolled. This study was performed in accordance with the Declaration of Helsinki and approved by the Ethics Committee of West China Stomatology Hospital, Sichuan University (No. WCHSIRB-D-2020-308). All patients signed an informed consent form. Patients fulfilling the criteria described below were recruited for this study. The workflow chart is shown in Fig. 1. All the surgeries were performed by the same surgeon.

The inclusion criteria were as follows: (1) head and neck tumor patients aged 20–70 years that were treated in our department; (2) required the lip-splitting approach during the operation; and (3) the patient consented to participate in the study.

The exclusion criteria were as follows: (1) patients with skin diseases that affect the recovery of surgical incisions and postoperative scar evaluation; (2) patients with wounds or scars on the lips or chin; and (3) history of the lip-splitting approach. Furthermore, those who revoked informed consent or did not follow the doctor's advice to review on time were removed from the study.

### Methodology

This study adhered to the CONSORT reporting guidelines.

#### Sample size

According to a previous study^10^, using an α of 0.05 and a power of 0.8, the required sample size to show a difference of two points was calculated to be at least 11 patients in each group. The sample size was increased to account for patients lost to follow-up due to COVID-19, and these patients were removed. In this study, at total of 49 patients were included in the final analysis.

#### Randomization

The RandomBetween function in Excel was used to generate 100 random integers between 1 and 10, and the numbers were plotted in a table. The table of random numbers was printed and each number was placed into an opaque envelope. For each included patient, the envelope was removed sequentially, opened before surgery by the surgeon in charge, and grouped according to parity. Patients, editors, and surgeons were blinded to which group the patient belonged to until the surgery started.

#### Data acquisition

1. Patient data: Patient’s clinical data were collected, including age, sex, diagnosis, pathology, systemic disease, surgical procedures (operation time, reconstruction, neck dissection, mandibulotomy, or mandibulectomy), recurrence, and adverse events (vascular crisis, infection, bleeding or hematoma, fistula, etc.).
2. Images: The patient's head-on, left oblique, and right oblique photos were taken 1 day before surgery and 7 days, 1 month, 3 months, and 6 months after the patient’s review. Each patient was photographed in a specific photo studio with a black background and a dedicated camera (M stop, shutter speed 1/100 s, aperture F10, ISO400), using a ring light.
3. Lip function evaluation: Lip function was evaluated using the Lip Function Assessment Scale, which contains eight items, each ranking on five levels. Higher scores indicated better function (Supplementary Table 1).
4. Scar evaluation: Scar formation was evaluated by the POSAS (Patient and Observer Scar Assessment Scale v2.0), with lower scores indicating better outcomes (Supplementary Tables 1–4).
5. Photographic analysis by naïve viewers: Patients’ head-on photographs at 6 months were viewed by three viewers who did not have any routine exposure to patients with head and neck cancer; photographs were assessed according to the naïve observer scar assessment scale (NOSAS; Supplementary Table 4).
6. Clinical examination: Notched vermilion, groove formation, and sensory disorders were assessed, as shown in Supplementary Table 5. Sensory disorder assessment was performed using a dental probe that stabbed normal skin and scar areas with the same strength.

#### Wound care and follow-up care

Patients are advised not to use topical drugs throughout the perioperative and follow-up periods, and not to use additional skin care products after wound healing. The study was stopped when sufficient samples were collected.

#### Statistical analysis

Ordinal data were analyzed using the Mann–Whitney U test. Categorical data were analyzed using the chi-squared test or Fisher’s exact test. Repeated measured data was analyzed using generalized estimating equations. Missing values occurred randomly due to patient dropout, and as their presence did not affect the analysis of Generalized Estimating Equations, no imputation was performed. Software used in the analysis were SPSS version 17.0 and GraphPad Prism version 5.0. A p-value < 0.05 was considered to be statistically significant.

#### Bias control

We mitigated potential risks through the application of randomization, the use of established assessment scales, and the establishment of stringent execution standards. Surgeon was unaware of the surgical procedure for each patient until they opened the envelope on the day of surgery. Postoperative evaluations were conducted by single, independent assessor. However, due to the visibility of the scar location, there was an inherent element of information bias. In the research process, we proactively contacted patients via telephone to remind them of their follow-ups, and those who were long-term lost to follow-up would be removed from the research.

### Surgical procedure

#### Straight incision

**Figure 2:**
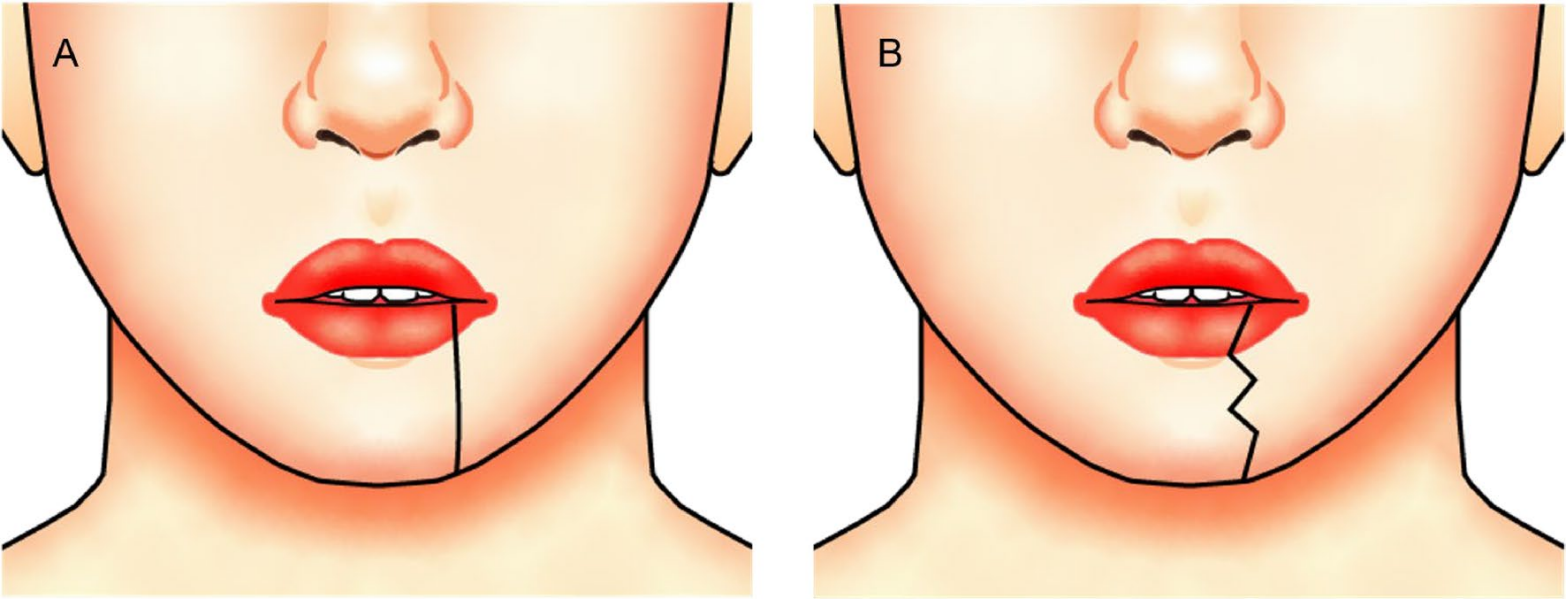
Straight incision (**A**) and Z-shaped incision (**B**).

The incision started from the vermilion border, moved down along a straight vertical incision, and was continued with a collar incision (Fig. 2A).

#### Z-shaped incision

The incision sloped down from the vermilion border to the lowest point of the mentolabial groove and turned backward toward the upper border of the chin protuberance. Another chevron was used to bypass the chin protuberance and was continued with a collar incision (Fig. 2B).

### Ethical approval

The work was approved by the Institutional Review Board of the West China Hospital of Stomatology, China (No. WCHSIRB-D-2020-308).

## Results

### Patient characteristics

The straight incision group consisted of 39 patients. Three patients were excluded because of a lip-splitting approach, two patients were excluded because they rejected the review or lost contact, three patients were excluded due to skin diseases, and 8 patients were excluded because they did not follow the doctors’ advice on time. Therefore, 23 patients were included in the final analysis. Among the 23 patients in the straight incision group, two patients (9%) experienced recurrence. Regarding adverse events, one patient (4%) developed a hematoma and one patient (4%) developed a submental fistula.

The Z-shaped incision group consisted of 49 patients. Two patients were excluded due to skin diseases, six patients were excluded due to postoperative radiotherapy implicated in the lower lip, and 15 patients were excluded because they did not follow the doctors’ advice on time. Thus, a total of 26 patients were included in the final analysis. All 26 patients in the Z-shaped incision group survived until the latest follow-up visit. Regarding adverse events, one patient (4%) experienced a vascular crisis and one patient (4%) experienced postoperative infection.

**Figure 3:**
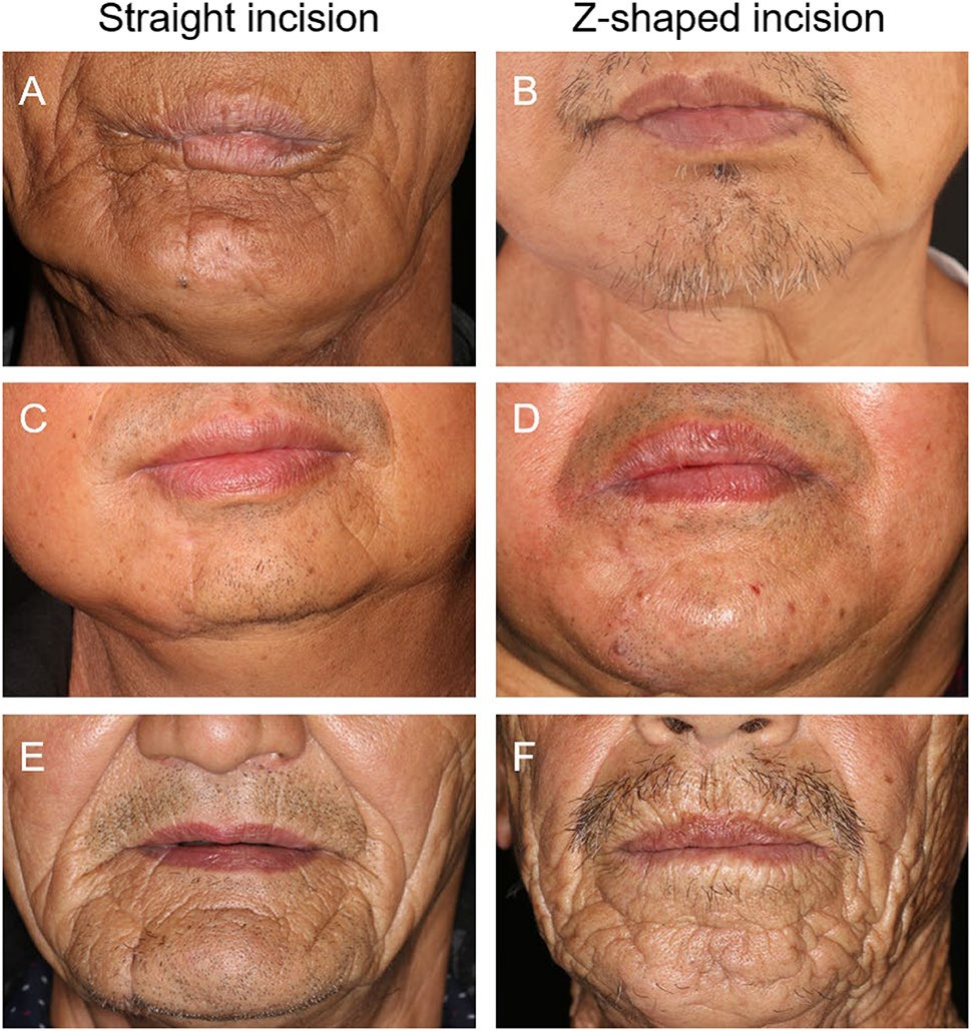
Digital image of patients who had undergone straight lower lip-splitting incision (**A**,**C**,**E**) and Z-shaped lower lip-splitting incision (**B**,**D**,**F**).

Both the groups had missing follow-up data. The detailed patient characteristics are shown in Table 1. Head-on views of patients from both groups are shown in Fig. 3.

**Table 1:** Patients profile.

Characteristics	No. of patients		P value
	Straight group (n = 23)	Z-shaped group (n = 26)	
Age	51.7 (± 14.6)	60.6 (± 13.6)	0.698^a^
< 60	11 (48%)	11 (42%)	
≥ 60	12 (52%)	15 (58%)	
Gender			0.674^a^
Male	18 (78%)	19 (73%)	
Female	5 (22%)	7 (27%)	
Primary tumor sites			0.502^b^
Tongue	9 (39%)	6 (23%)	
Oropharynx	3 (13%)	3 (11%)	
Gingiva	1 (4%)	5 (19%)	
Floor of mouth	3 (13%)	3 (11%)	
Mandible	1 (4%)	0 (0%)	
Buccal	6 (26%)	9 (35%)	
Pathology			0.469^b^
SCC	22 (96%)	26 (100%)	
PIOC	1 (4%)	0 (0%)	
T stage			0.300^a^
T2	11 (49%)	10 (39%)	
T3	4 (17%)	10 (39%)	
T4	8 (35%)	6 (23%)	
N stage			0.223^b^
N0	4 (17%)	10 (39%)	
N1	6 (26%)	8 (31%)	
N2	9 (39%)	4 (15%)	
N3	4 (17%)	4 (15%)	
Overall stage			0.742^b^
I	4 (17%)	4 (15%)	
II	7 (30%)	11 (42%)	
III	12 (52%)	11 (42%)	
Systemic disease			0.620^b^
Hypertension	4 (17%)	7 (27%)	
DM	1 (4%)	1 (4%)	
Hepatitis B	1 (4%)	0 (0%)	
Hyperthyroidism	1 (4%)	0 (0%)	
RA	1 (4%)	0 (0%)	
SS	1 (4%)	0 (0%)	

P-value represent Fisher’s exact test. *SCC* squamous cell carcinoma, *PIOC* primary intraosseous carcinoma, *DM* diabetic mellitus, *RA* rheumatoid arthritis, *SS* Sjogren's syndrome.

^a^Represent chi-square test.

^b^Represent Fisher exact test.

### Evaluation of postoperative lip competence

**Figure 4:**
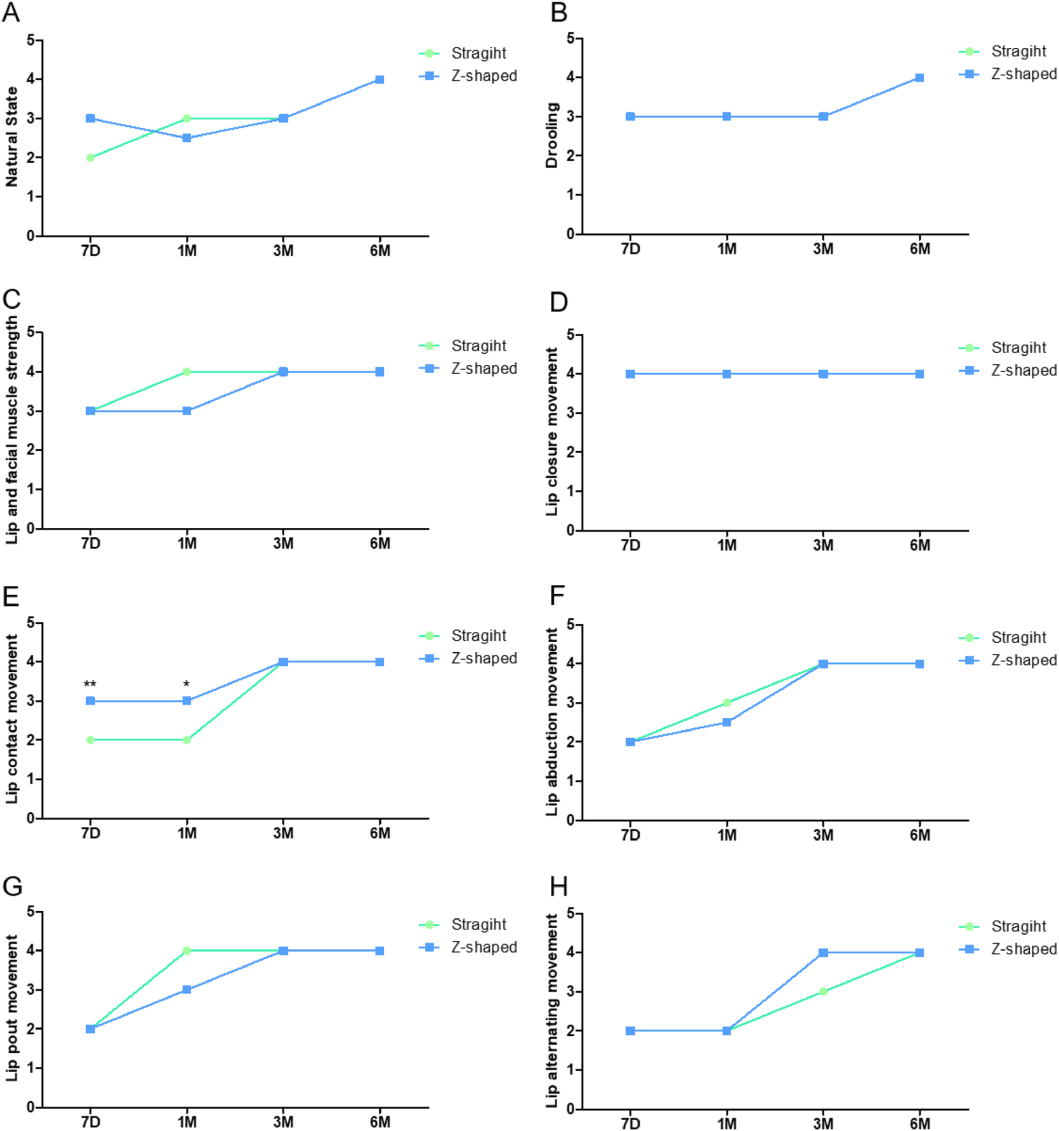
Lip function assessment scale result. *Represents P < 0.05. **Represents P < 0.01.

We followed up the patients and analyzed the data based on time points. As shown in Fig. 4 (a higher score indicates better function), the Z-shaped incision group performed better than the straight incision group. The total scores on the lip function assessment scale also showed significant difference between the two groups (Table 2).

**Table 2:** Scar assessment scale scores.

Assessment*	Straight group	Z-shaped group	P value
LFAS			
7D	20.3(± 3.3) (21,12–26)	22.7(± 3.7) (21,14–27)	0.025^a^
1M	21.4(± 3.7) (21,17–27)	23.7(± 4.2) (22,17–29)	
3M	23.5(± 4.5) (24.5,17–29)	27.9(± 3.3) (27,20–31)	
6M	28.4(± 3.6) (30,20–32)	27.6(± 4.9) (29.5,21–32)	
PSAS			
7D	26.1(± 4.0) (25,22–39)	26.9(± 4.7) (27,20–37)	0.287^a^
1M	29.8(± 3.2) (29.5,25–35)	30.5(± 9.5) (25,16–44)	
3M	17.1(± 3.8) (17,13–26)	17.7(± 4.3) (16,11–24)	
6M	13.9(± 2.6) (14,11–22)	10.7(± 3.0) (11,7–15)	
OSAS			
7D	24.5(± 4.2) (24,19–37)	22.8(± 3.8) (23,18–35)	0.066^a^
1M	27.7(± 5.6) (26,22–38)	25.0(± 4.3) (24.5,16–33)	
3M	15.5(± 4.0) (15,10–29)	14.5(± 3.3) (14,11–24)	
6M	13.4(± 3.6) (13,8–26)	11.3 (± 3.4) (10.5,8–17)	
NOSAS			
6M	8.0(± 2.0) (8,5–15)	6.85(± 2.0) (6.5,4–10)	0.013^b^

*Data are given as mean (SD) (median, range).

^a^P-value represent GEE.

^b^P-value represent Mann–Whitney test.

### Evaluation of postoperative scar formation

**Figure 5:**
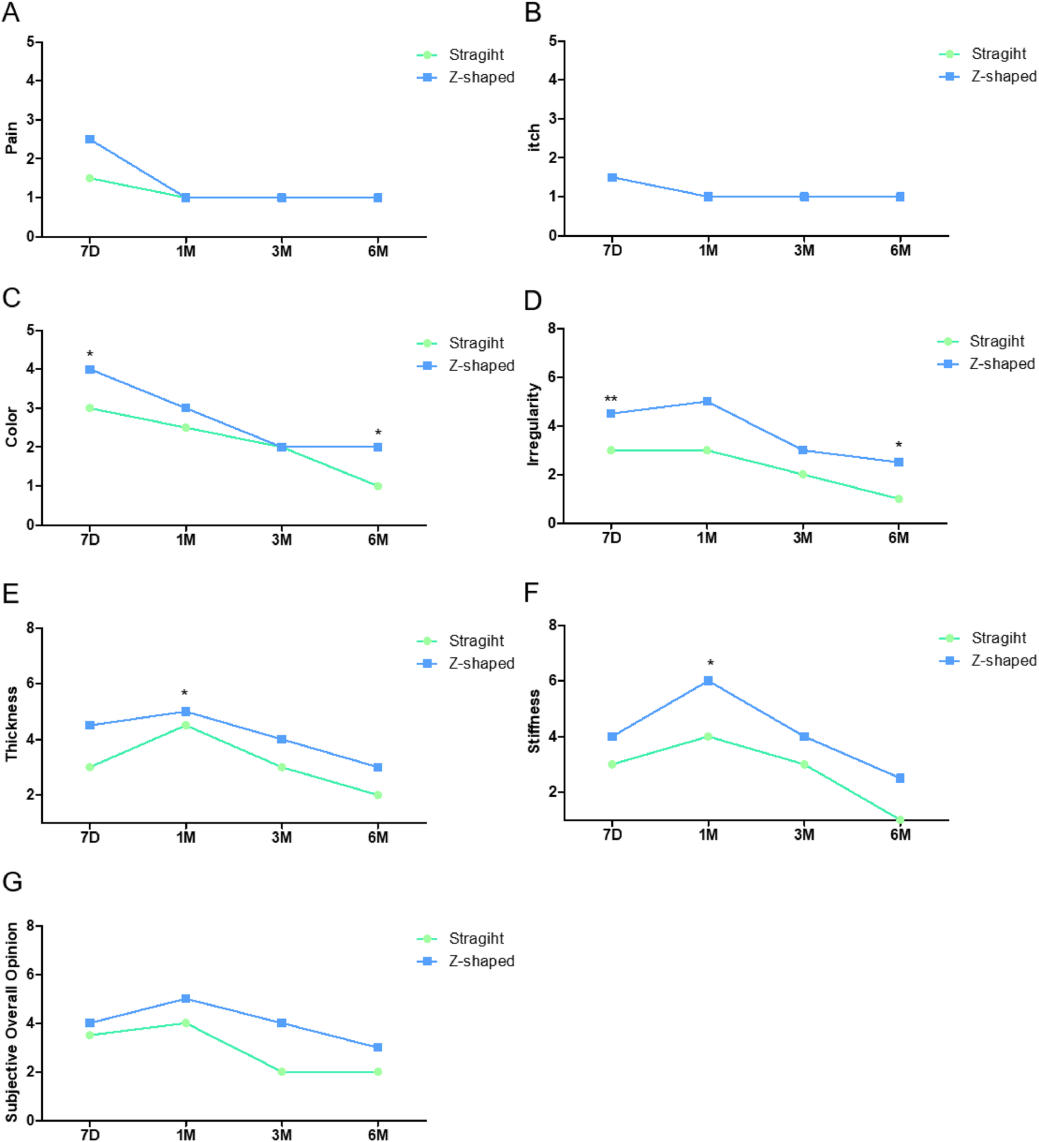
Patient scar assessment scale results. *Represents P < 0.05. **Represents P < 0.01.

**Figure 6:**
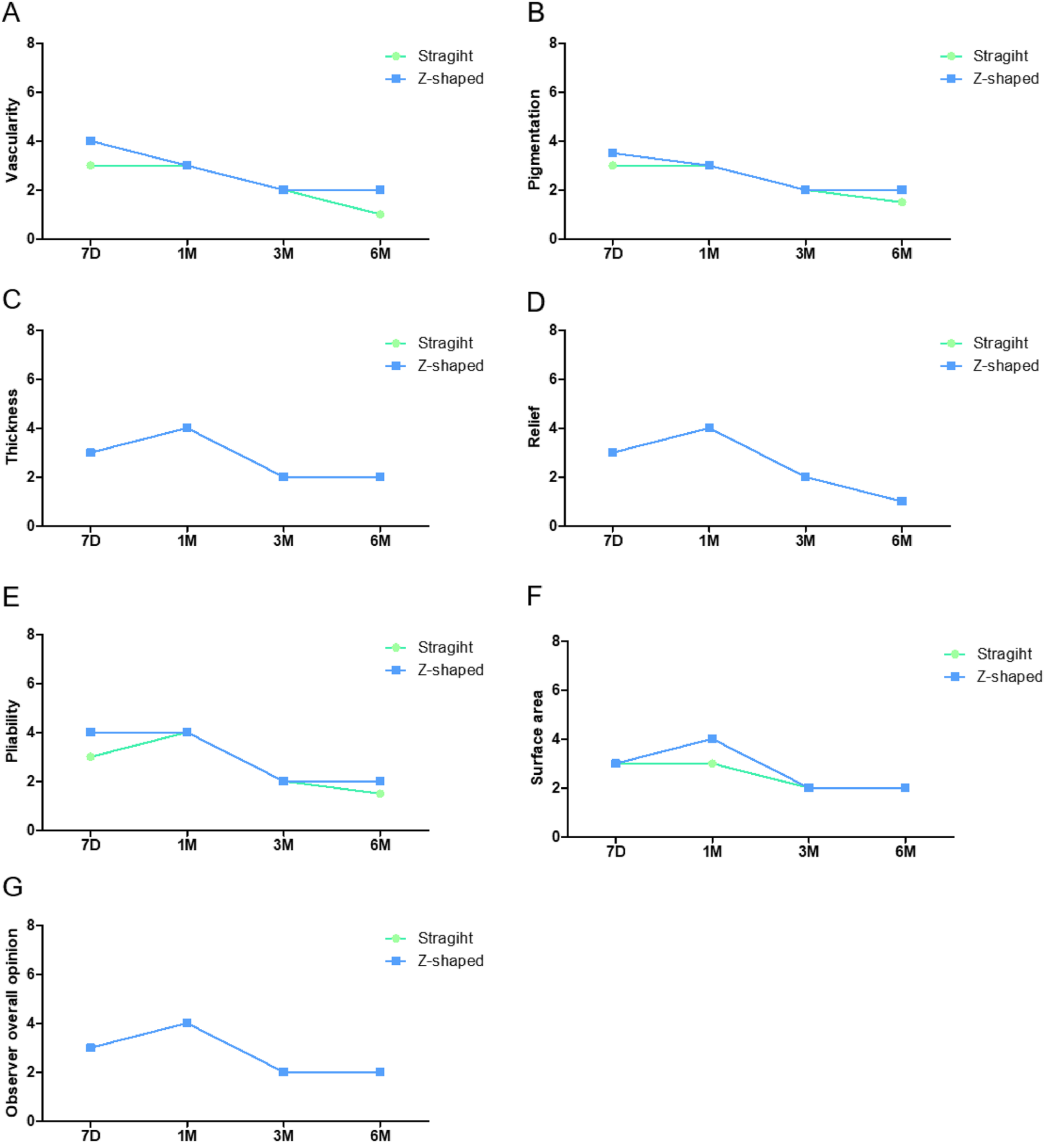
Observer scar assessment results.

The total PSAS scores didn’t show significantly different between the Z-shaped and straight incision groups (Table 2, Fig. 5). Regarding the OSAS, the total scores didn’t show significant differences between the two groups either (Fig. 6). There was a bias in the study because the study design did not blind the assessors. Therefore, to obtain credible results, we used the NOSAS and found that the results showed significant difference (Table 2).

### Evaluation of clinical examination

We further assessed notched vermilion, groove formation, and sensory disorders that were not considered in the POSAS and NOSAS (Table 3). As expected, the notched vermilion incidence was significantly lower in the Z-shaped group, while groove formation did not show a significant difference. Regarding sensory disorders, most patients (85%) showed noticeably better performance at 6 months than at 7 days; however, there was no significant difference between the two groups.

**Table 3:** Clinical examination.

Assessment	Straight group (n = 23)			Z-shaped group (n = 26)			P value
Notched vermillion	1	2		1	2		
7D	0 (0%)	22 (96%)		0 (0%)	25 (96%)		0.002
1M	9 (39%)	2 (9%)		1 (4%)	19 (73%)		
3M	12 (52%)	9 (39%)		6 (23%)	19 (73%)		
6M	10 (44%)	13 (57%)		6 (23%)	20 (77%)		

Groove formation	1	2		1	2		
7D	0 (0%)	22 (96%)		6 (23%)	19 (73%)		0.185
1M	10 (44%)	1 (4%)		11 (42%)	9 (34%)		
3M	12 (52%)	9 (39%)		19 (73%)	6 (23%)		
6M	10 (44%)	13 (57%)		14 (53%)	12 (46%)		

Sensory disorder	1	2	3	1	2	3	
7D	22 (96%)	0 (0%)	0 (0%)	25 (92%)	0 (0%)	0 (0%)	0.229
1M	10 (44%)	1 (4%)	0 (0%)	19 (73%)	1 (4%)	0 (0%)	
3M	15 (65%)	6 (26%)	0 (0%)	14 (54%)	9 (35%)	2 (7.7%)	
6M	5 (22%)	13 (61%)	4 (17%)	1 (4%)	20 (77%)	5 (20%)	

Notched Vermillion: 1. Yes, 2. No; Groove formation: 1. Yes, 2. No; Sensory disorder: 1. anesthesia, 2. dysesthesia, 3. normal. P-value represent GEE.

### Other possible factors

In the case of other potentially affecting factors, we further collected operation variables, such as operation time, reconstruction type, neck dissection type, and mandible disposal (Supplementary Table 6). Multivariate logistic regression was used to analyze the relationship between the operational variables and the total scores of the scales; no significant differences were found.

## Discussion

In this study, the novel Z-shaped incision was found to have significantly better functional and aesthetic outcomes than the traditional straight incision, suggesting that the Z-shaped incision may be used as an alternative to the traditional vertical incision for conventional lower lip incisions. There are some limitations to this study: firstly, our study was a single-center study, the relatively small sample size collected might limit the statistical power of the study; and it may not generalize to a broader population due to potential selection biases in patient enrollment; and constraints in resources and expertise could have led to limitations in the study design, data collection, and analysis. These aspects should be considered in the interpretation of the findings. Secondly, Due to financial and time constraints, as well as the nature of the research design, our sample size and follow-up time have obvious disadvantages compared to other retrospective studies, which may affect the statistical power of the study, making it difficult to draw definitive conclusions; further expansion of the sample size and follow-up time could provide a more solid basis for our study conclusions. Third, as psychological impact assessment is an important part of quality of life, it is necessary to explore the psychological impact of scar appearance and lip function on patients, which may add important dimensions to this study. Finally, because the above-mentioned modified incisions have not been widely used in our hospital, the experimental design cannot simultaneously compare the functional and aesthetic outcomes of multiple incisions at the same time. The addition of other modified incisions as a control group will undoubtedly better prove the effect of the Z-shaped incision; this part of the study will be considered in future experiments.

The design of the Z-shaped incision is similar to that of Hayter’s incision but uses a larger triangle flap to achieve a better tension-relaxing effect and reduces ischemia. Meanwhile, we changed the arc-shaped incision to a triangular flap to avoid the implication of chin protuberance; the results showed that it fits the contour of the chin protuberance well. One of the initial intentions of the Z-shaped incision design was to reduce oral incontinence or drooling by reducing the number of notched vermilions. However, the results showed that the notched vermilion is not the most important factor influencing food and saliva incontinence. Although oral incontinence or drooling was a common problem among our patients during their hospital stay, our results showed that most of our patients got used to it and controlled it well when assessed 1 month later. As far as our clinical experience is concerned, oral incontinence or drooling in our patients could be the result of three other factors: (1) impaired tongue and lip motility caused by free flap transplantation and scarring preventing patients from stirring food evenly and closing the mouth timely; (2) decreased oral capacity due to free flap transplantation, requiring the patient to swallow more frequently than usual, especially when the lesion implicated the floor of the mouth or gingiva; and (3) loss of tongue, oral mucosa, and lip sensation caused by ablative surgery, tissue edema, or injury to the inferior alveolar nerve prevented the patient from feeling the drool and food that was about to flow out. Although oral mucosa and tongue sensation loss caused by ablative surgery could not be recovered, as observed in this study, lip sensation in most patients improved after 6 months. According to incomplete statistics, most patients can completely recover the sensation of the lower lip approximately 1 year after surgery. Furthermore, because the rank data in this study were not clear enough to clarify the process of lip sensation recovery after lower lip incision, more detailed and objective research is ongoing. Our research indicates that the Z-shaped incision promotes faster recovery of lip function following surgery, enabling patients to adapt more swiftly and regain control over their post-operative eating activities. This, in turn, supports better nutritional intake, contributing to an overall faster physical rehabilitation after the procedure.

Based on clinical experience, straight line incisions can be aesthetically acceptable when precisely sutured. However, by comparing the best aesthetic results of each group, we found that the straight group inevitably left some marks, while the Z-shaped group sometimes showed difficulty in finding the incision scar at first sight. The disguising effect of beards and wrinkles on the incision scars was better in the Z-shaped incision group. Improved facial aesthetics essentially translates to reduced stress for patients post-surgery, stemming from issues like damaged self-esteem, social difficulties, or even occupational impacts, thereby ensuring a higher quality of life post-procedure. Given the favorable outcomes observed, this study should be conducted and expanded upon in more research institutions to derive more universally applicable conclusions and further enhance patients' postoperative quality of life.

In conclusion, this study revealed the process of lip function recovery and scar formation 6 months after a lower lip-splitting incision. The Z-shaped lower lip-splitting incision was deemed to be as safe as the straight incision, with a faster recovery and a better aesthetic effect.

### Supplementary Information

Supplementary Information.

## Data Availability

Contact the first or corresponding author for data and materials.
