# Supplementary material for: Functional and aesthetic results of the Z-shaped and straight lower lip-splitting incision: a randomized clinical trial
Source: Sci Rep. 2024 Aug 12;14:18699. doi: 10.1038/s41598-024-63983-z (PMC11319339; doi:10.1038/s41598-024-63983-z)
Supplement: Supplementary file 1 — Supplementary Information. [file 41598_2024_63983_MOESM1_ESM.docx]

Supplementary Table 1. Lip function assessment scale

| Dimension | Grading (higher score indicating better function) |
| --- | --- |
| 1.Natural state | |
| 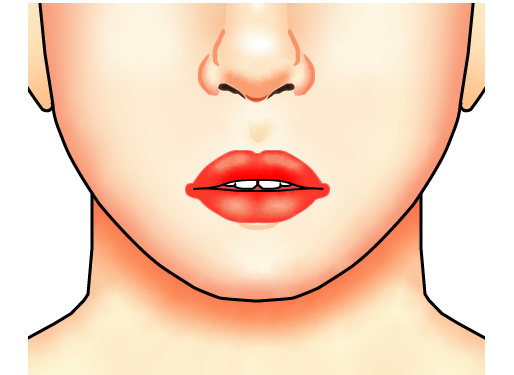 | 〇level 0：The lips are severely asymmetrical and the position is almost unchanged. |
|  | 〇level 1：Upper or lower lip retraction is severe, and the upper or lower lip is shaken when relaxed, but the patient does not realize. |
|  | 〇level 2：The upper or lower lip has a slight tremor when relaxed, but the patient occasionally tries to reset; or asymmetrical lips. |
|  | 〇level 3：Slight retraction of the upper lip or slight retraction of the lower lip, or slight misalignment, not obvious. |
|  | 〇level 4：The lips are naturally in the middle of the horizontal position, symmetrical, and naturally closed. |
| 2.Drooling： | |
| 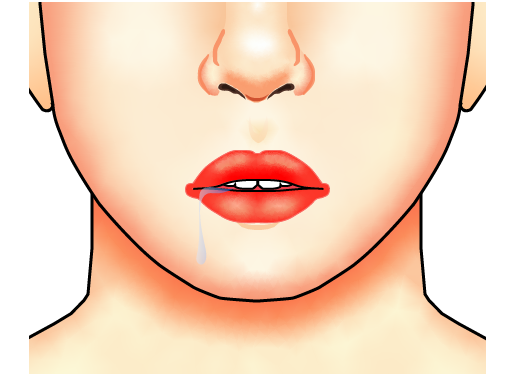 | 〇level 0：Out of control. |
|  | 〇level 1：Drool when the body leans forward or when distracted, slightly controlled. |
|  | 〇level 2：Just drool at the corners of the mouth and can be controlled slightly. |
|  | 〇level 3：The corners of the mouth are damp occasionally or slightly drool when drinking water or chewing. |
|  | 〇level 4：No drooling. |
| 3.Lip and facial muscle strength examination： | |
| 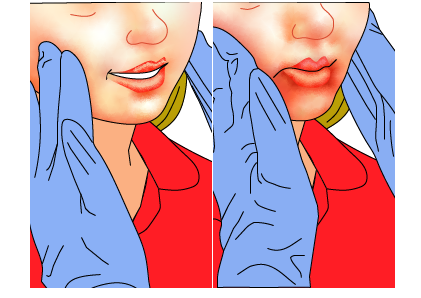 | 〇level 0：Refuse to do it. |
|  | 〇level 1：Cheeks are stiff, or keep smiling face, or muscles are tightly contract, making it difficult to make a grimace. |
|  | 〇level 2：Cheek muscles are soft, or easy to make a grimace. |
|  | 〇level 3：Upper lip retraction or lower lip retraction. |
|  | 〇level 4：The cheeks are relaxed at rest and the muscle strength is normal. |
| 4.Lip closure movement： | |
| 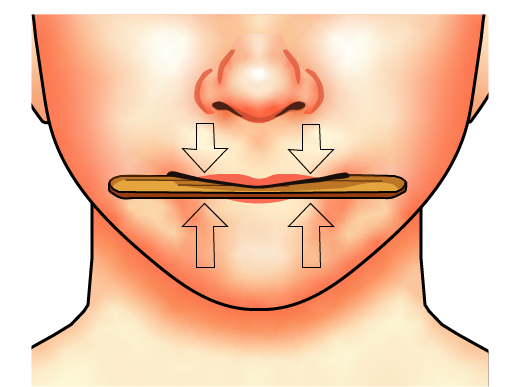 | 〇level 0: No reaction |
|  | 〇level 1: Lips can contact each other, try to pinch the tongue depressor but fail to hold it, or only can bite it with teeth. |
|  | 〇level 2: Effort is required when lips are tightly closed; Or Lips cannot clamp the tongue depressor more than 1 second. |
|  | 〇level 3：The lips can clamp the tongue depressor and but last no more than 3 seconds. |
|  | 〇level 4：The lips can clamp tongue depressor tightly and last more than 3 seconds. |
| 5.Lip contact movement： | |
| 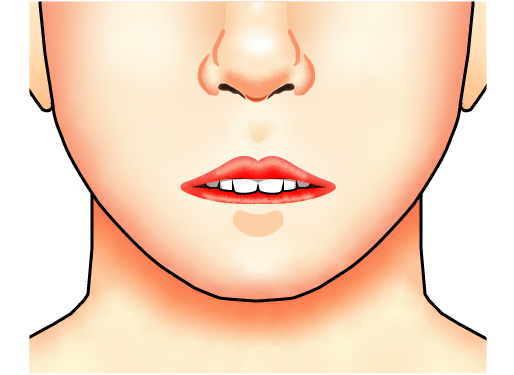 | 〇Level 0：No reaction |
|  | 〇Level 1: Try to abduce or pout the lip, but cannot finish, compensate or assist with eyes, head, or shoulders. |
|  | 〇Level 2：The upper teeth cannot bite the back of the lower lip, but they can bite the lower lip. |
|  | 〇Level 3: The upper teeth can bite the back of the lower lip but last no more than 3 seconds. |
|  | 〇Level 4: The upper teeth can bite the back of the lower lip and last more than 3 seconds. |
| 6.Lip abduction movement： | |
| 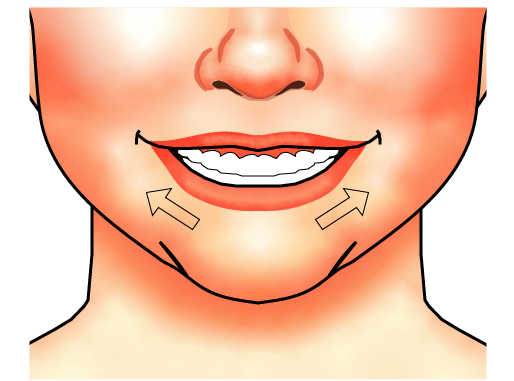 | 〇Level 0: No reaction. |
|  | 〇Level 1: Try to abduce, but cannot finish, compensate or assist with eyes, head, or shoulders. |
|  | 〇Level 2: Effort is required to abduce Lips, or the corner of the mouth cannot be raised; Or a small range of abduction, or stiffness or weakness in abduction |
|  | 〇Level 3: Lips can abduce but last no more than 3 seconds. |
|  | 〇Level 4: Lips can fully abduce, grin and last more than 3 seconds. |
| 7.Lip pout movement： | |
| 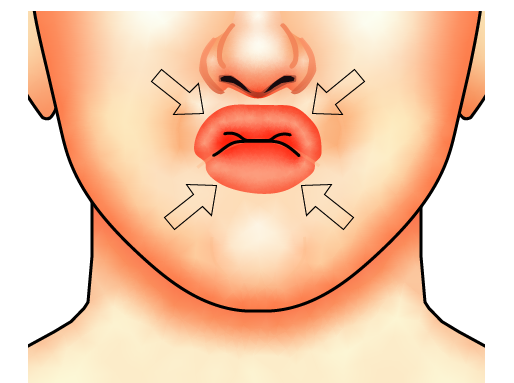 | 〇Level 0: No reaction. |
|  | 〇Level 1: Try to pout, but cannot finish, compensate or assist with eyes, head, or shoulders. |
|  | 〇Level 2: Effort is required to pout Lips; Or a small range of pout, or stiffness or weakness in pout. |
|  | 〇Level 3: Lips can pout but last no more than 3 seconds. |
|  | 〇Level 4: Lips can fully pout and last more than 3 seconds. |
| 8. Lip abduction and pout alternating movement： | |
|  | 〇Level 0: No reaction. |
|  | 〇Level 1: Try to abduce or pout, but cannot finish, compensate or assist with eyes, head, or shoulders. |
|  | 〇Level 2: Can only finish one of abduction or pout movement; Or a small range of abduction or pout, or stiffness or weakness in abduction or pout. |
|  | 〇Level 3: Lips can continuously abduce and pout but no more than 3 times. |
|  | 〇Level 4: Lips can continuously abduce and pout more than 3 times. |


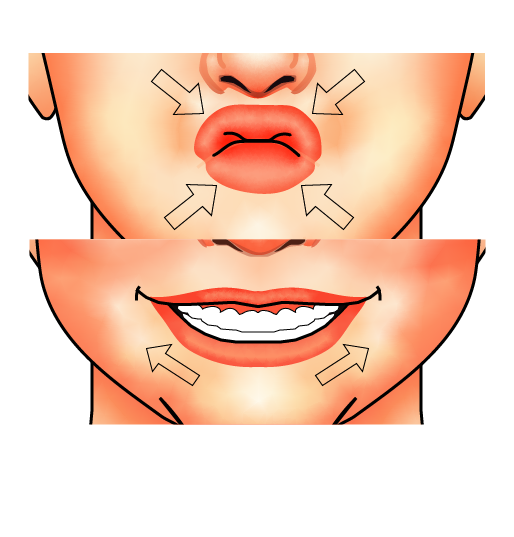
Supplementary Table 2. Patients scar assessment scale

|  | 1=no，not at all | | | | | yes，very much=10 | | | | |
| --- | --- | --- | --- | --- | --- | --- | --- | --- | --- | --- |
|  | 1 | 2 | 3 | 4 | 5 | 6 | 7 | 8 | 9 | 10 |
| Has the scar been painful in the past two weeks? | ○ | ○ | ○ | ○ | ○ | ○ | ○ | ○ | ○ | ○ |
| Has the scar been itching in the past two weeks? | ○ | ○ | ○ | ○ | ○ | ○ | ○ | ○ | ○ | ○ |
| Is the scar color different from the color of your normal skin at present? | ○ | ○ | ○ | ○ | ○ | ○ | ○ | ○ | ○ | ○ |
| Is the stiffness of the scar different from your normal skin at present? | ○ | ○ | ○ | ○ | ○ | ○ | ○ | ○ | ○ | ○ |
| Is the thickness of the scar different from your normal skin at present? | ○ | ○ | ○ | ○ | ○ | ○ | ○ | ○ | ○ | ○ |
| Is the scar more irregular than your normal skin at present? | ○ | ○ | ○ | ○ | ○ | ○ | ○ | ○ | ○ | ○ |
| What is your overall opinion of the scar compared to normal skin? | ○ | ○ | ○ | ○ | ○ | ○ | ○ | ○ | ○ | ○ |

Supplementary Table 3. Observer scar assessment scale

|  | 1=Normal skin | | | | | Worst scar imaginable=10 | | | | |  |
| --- | --- | --- | --- | --- | --- | --- | --- | --- | --- | --- | --- |
| Parameter | 1 | 2 | 3 | 4 | 5 | 6 | 7 | 8 | 9 | 10 | Category |
| Vascularity | ○ | ○ | ○ | ○ | ○ | ○ | ○ | ○ | ○ | ○ | Pale/Pink/Red/Purple/Mix |
| Pigmentation | ○ | ○ | ○ | ○ | ○ | ○ | ○ | ○ | ○ | ○ | Hypo/Hyper/Mix |
| Thickness | ○ | ○ | ○ | ○ | ○ | ○ | ○ | ○ | ○ | ○ | Thicker/Thinner |
| Relief | ○ | ○ | ○ | ○ | ○ | ○ | ○ | ○ | ○ | ○ | More/Less/Mix |
| Pliability | ○ | ○ | ○ | ○ | ○ | ○ | ○ | ○ | ○ | ○ | Supple/Stiff/Mix |
| Surface area | ○ | ○ | ○ | ○ | ○ | ○ | ○ | ○ | ○ | ○ | Expansion/Contraction /Mix |
| Overall opinion | ○ | ○ | ○ | ○ | ○ | ○ | ○ | ○ | ○ | ○ |  |

Explanation：

The observer scale of the POSAS consists of six items (vascularity, pigmentation, thickness, relief, pliability and surface area).

All items are scored on a scale ranging from 1(‘like normal skin’) to 10(‘worst scar imaginable’)

The sum of the six items results in a total score of the POSAS observer scale. Categories boxes are added for each item. Furthermore, an overall opinion is scored on a scale ranging from 1 to 10.

All parameters should preferably be compared to normal skin on a comparable anatomic location.

Explanatory notes on the items:

vascularity: Presence of vessels in scar tissue assessed by the amount of redness, tested by the amount of blood return after blanching with a piece of Plexiglas.

Pigmentation: Brownish coloration of the scar by pigment(melanin); apply Plexiglas to the skin with moderate pressure to eliminate the effect of vascularity.

Thickness: Average distance between the subcutical-dermal border and the epidermal surface of the scar.

Relief: The extent to which surface irregularities are presented (preferably compared with adjacent normal skin).

Pliability: Suppleness of the scar tested by wrinkling the scar between the thumb and index finger.

Surface Area: Surface area of the scar in relation to the original wound area.

Supplementary Table 4. Naïve Observer Scar Assessment Scale

| Normal skin | 1 | | 2 | | 3 | | 4 | | 5 | | 6 | | 7 | | 8 | | 9 | | 10 | | Worst scar imaginable |
| --- | --- | --- | --- | --- | --- | --- | --- | --- | --- | --- | --- | --- | --- | --- | --- | --- | --- | --- | --- | --- | --- |
| Is the color of the lip-splitting scar different? | ○ | ○ | | ○ | | ○ | | ○ | | ○ | | ○ | | ○ | | ○ | | ○ | |  | |
| Is the contour of the lip-splitting scar different? | ○ | ○ | | ○ | | ○ | | ○ | | ○ | | ○ | | ○ | | ○ | | ○ | |  | |
| Is the lip-splitting scar distorted? | ○ | ○ | | ○ | | ○ | | ○ | | ○ | | ○ | | ○ | | ○ | | ○ | |  | |
| Does the patient appear disfigured from his/her facial scars? | ○ | ○ | | ○ | | ○ | | ○ | | ○ | | ○ | | ○ | | ○ | | ○ | |  | |

Supplementary Table 5. Clinical examination

| Assessment | Category | Explanation |
| --- | --- | --- |
| Notched vermillion | 1 | A notch is visible from vermillion border |
|  | 2 | Continuous vermillion border without notch |
| Groove formation | 1 | A groove is visible from vermillion to chin |
|  | 2 | Continuous skin surface without groove |
| Sensory disorder | 1 | Anesthesia |
|  | 2 | Dysesthesia |
|  | 3 | Normal |

Supplementary Table 6. Operation profile

| Variables | Straight (n=23) | Z-shaped (n=26) | P value |
| --- | --- | --- | --- |
| Operation time (min) | 252.3 (±66.4) | 258.6 (±63.3) | 0.841^a^ |
| Reconstruction |  |  | 0.034^b^ |
| ALTFF | 21 (91%) | 20 (77%) |  |
| LDMCF | 0 (0%) | 2 (8%)) |  |
| LAFF | 2 (9%) | 0 (0%)) |  |
| RFFF | 0 (0%) | 4 (15%) |  |
| Neck dissection |  |  | 0.302^b^ |
| Unilateral RND | 12 (52%) | 17 (65%) |  |
| Bilateral RND | 10 (44%) | 6 (23%) |  |
| Unilateral SND | 1 (4%) | 3 (12%) |  |
| Mandible |  |  | 0.158^b^ |
| Mandibulotomy | 6 (26%) | 3 (12%) |  |
| Marginal mandibulectomy | 16 (70%) | 18 (69%) |  |
| Segmental mandibulectomy | 0 (0%) | 4 (15%) |  |

^a^ P-values represents t test, ^b^ P-value represent Fisher’s exact test. *ALTFF* anterolateral thigh perforator free flap, *LDMCF* latissimus dorsi myocutaneous free flap *LAFF* lateral arm free flap, *RFFF* radial forearm free flap, *RND* radical neck dissection, *SND* selective neck dissection.
